# Influence of folic acid knowledge on effective folic acid intake in Chinese pregnant women: a cross-sectional study

**DOI:** 10.4069/kjwhn.2023.11.20

**Published:** 2023-12-28

**Authors:** You Jing Jin, Hae Won Kim

**Affiliations:** 1 College of Nursing, Seoul National University, Seoul, Korea; 2 The Research Institute of Nursing Science Seoul National University, Seoul, Korea; 3 Center for Human-Caring Nurse Leaders for the Future by Brain Korea 21 (BK 21) Four Project, Seoul National University, Seoul, Korea

**Keywords:** Folic acid, Knowledge, Neural tube defects, Pregnant women, Women’s health

## Abstract

**Purpose:**

This study aimed to investigate the current status of effective folic acid intake and the level of folic acid knowledge of Chinese pregnant women and to analyze the relationship between effective folic acid intake and folic acid knowledge.

**Methods:**

From November 2021 to May 2022, 140 pregnant women at Yantai Yuhuangding Hospital in the Chinese province of Shandong, answered questions about their general characteristics, folic acid intake, and folic acid knowledge. The data were analyzed using the t-test, chi-square test, and logistic regression analysis, and were presented with frequency with percentage or mean±standard deviation.

**Results:**

Only 16.4% of the pregnant women (n=23) took folic acid effectively, using the following four criteria. Of all pregnant women who took folic acid, 72.2% took folic acid starting 1 month before pregnancy, 70.8% took folic acid up to 3 months after pregnancy, 36.8% took 400 μg every time, and 78.6% took folic acid more than 24 days every month. The score for folic acid knowledge was relatively high (5.61±2.18 on a scale of 0–9). A higher folic acid knowledge score correlated with more effective folic acid intake (t=4.10, *p*<.001).

**Conclusion:**

Our study shows that the current recommendations to prevent neural tube defects through effective folic acid intake supplementation are not being fully implemented in China. Furthermore, folic acid knowledge was positively correlated with the effectiveness of its intake. Future education related to effective folic acid intake should emphasize the four methods of effective folic acid intake, especially regarding the recommended dose of 400 μg every time.

## Introduction

According to guidelines from the Centers for Disease Control and Prevention (CDC), all women with the potential to bear children should consume 400 µg of folic acid daily, starting at least 1 month before pregnancy and continuing until 3 months into pregnancy. This is to prevent fetal neural tube defects, including spondyloschisis, anencephaly, and cephalocele [1]. The prevalence of neural tube defects varies across regions as defined by the World Health Organization. A systematic review conducted in 2016 reported incidences of 21.9 per 10,000 people in the East Mediterranean region, 15.8 in Southeast Asia, 11.7 in Africa, 11.5 in the Americas, 9.0 in Europe, and 6.9 in the West Pacific region [2]. With its vast size, China exhibits varying incidences of neural tube defects across different regions. Higher rates are observed in the northern and western parts, while lower rates are seen in the southern and eastern regions. Although the average incidence in Mainland China is 6.18 per 10,000, a closer look at each region reveals the following: in North China, the Inner Mongolia Autonomous Region has an incidence of 20.1 per 10,000, and Shanxi Province has 16.07 per 10,000. In East China, Anhui Province has 10.65 per 10,000. Northwest China has an average incidence of 20 per 10,000, with Gansu Province showing an incidence of 39.51 per 10,000. These findings can be attributed to factors such as the ecological environment, economic development, and the standard of the healthcare system [3]. There appears to be a need for studies that identify the factors influencing the reduction of neural tube defect incidence, with the aim of mitigating their negative effects.

While the exact cause of neural tube defects remains unknown, a deficiency in folic acid during pregnancy is considered a significant factor. Research indicates that consuming folic acid during pregnancy can prevent between 50% and 70% of fetal neural tube defects [4]. Despite global initiatives promoting the prevention of neural tube defects through adequate folic acid intake, a study by Chitayat et al. [5] suggests that the rate of effective folic acid consumption is low, resulting in insufficient prevention of these defects. Effective folic acid intake is defined as the prevention of neural tube defects in women of childbearing age by consuming 400 µg of folic acid, starting at least 1 month before pregnancy and continuing until 3 months after pregnancy [1]. In this study, effective folic acid intake was specified as follows: (1) a daily intake of 400 µg of folic acid, (2) beginning 1 month prior to pregnancy, (3) continuing until 3 months post-pregnancy, and (4) when the correct intake of folic acid occurs on 80% or more of the total number of days [1,6].

Most previous studies on the four criteria for effective folic acid consumption in various countries did not clearly define the exact period of intake. In studies carried out in China, it was found that between 7.9% and 32.7% of women began taking folic acid prior to pregnancy [7,8], 55.7% started 2 months post-pregnancy [7], and 14.3% took it from 3 months pre-pregnancy until 3 months post-pregnancy [9]. In terms of folic acid intake prior to pregnancy in other countries, 20.5% of Japanese women began 1 month before pregnancy [10], and 11.7% of Italian women did the same [11]. In Korea, 24.6% of women started taking folic acid before pregnancy, regardless of the specific period [12], while 24.7% of Irish women [13] and 30% of American women [14] followed suit. In contrast, between 54.9% and 74.9% of Italian women began their folic acid intake after pregnancy [11,15], and 47.2% of Italian women took a daily dose of 400 µg [15]. These findings illustrate the patterns of folic acid consumption among pregnant women in Northwest China and other parts of the world. Few studies, whether in China or elsewhere, have specified the exact period of intake. The rate of intake before pregnancy was relatively low, with most participants beginning their folic acid regimen after becoming pregnant. While previous studies have reported the rate of folic acid intake at various stages before and after pregnancy, few have addressed the overall effective intake rate. Therefore, this study aimed to investigate the extent to which the criteria for effective intake are being met by pregnant women in China. Furthermore, it is crucial to provide Chinese women with education on proper folic acid consumption to improve their understanding of effective folic acid intake and encourage its use.

Folic acid knowledge refers to the awareness that folic acid belongs to the vitamin B complex, and that an adequate amount of folic acid in the body can help prevent significant congenital defects in the fetal brain and spine. It also involves knowing that a daily dose of 400 µg of folic acid is necessary from 1 month before pregnancy until 3 months after pregnancy [1]. Existing research on folic acid knowledge reveals that 58.3% of pregnant women in China were aware that folic acid intake is necessary to prevent neural tube defects. However, only 15.6% knew the correct intake period for folic acid, and 36.7% knew the accurate dosage [16]. Studies have shown that 56.4% of pregnant women in Korea and 85.4% in Ireland were aware that folic acid helps prevent neural tube defects [12,17]. Additionally, 32.2% of pregnant women in Japan knew the exact dosage of folic acid [10]. In summary, the level of folic acid knowledge among pregnant women in China appears to be similar to that of pregnant women in Korea, particularly regarding folic acid’s role in preventing neural tube defects. It is also comparable to the knowledge level among pregnant women in Japan concerning the dosage of folic acid. Although there was no comparison group for the intake period of folic acid, it was found that few women had accurate knowledge of this aspect.

An analysis was conducted to determine the correlation between knowledge of folic acid and its intake. The results revealed that pregnant women who understood the role of folic acid in preventing neural tube defects were 2.64 times more likely to consume folic acid compared to those who were unaware of its benefits [10]. This suggests that simply being aware of the need for folic acid can influence its actual consumption. Given that accurate knowledge about folic acid can potentially enhance its effective intake, this study aimed to further investigate this correlation.

Upon investigating other factors that influence folic acid intake, it was observed that in both China and other countries, certain factors were associated with a higher rate of folic acid consumption. These factors included being aged 30 years and above [10,13,18], having a higher level of education [18-20], earning a higher income [20,21], residing in cities [19], suffering from chronic diseases [11,22], planning pregnancies [19,22], being a married woman [11,21], having given birth [11,13,21], and having undergone infertility treatment.

A review of the above references shows that most existing studies have only incorporated certain criteria when defining effective folic acid intake. These studies have investigated the current state of folic acid consumption and analyzed its alignment with the understanding of folic acid. Therefore, this study aimed to evaluate the status of effective folic acid intake among pregnant women in China, in line with the four guidelines issued by the CDC. Furthermore, the study investigated women’s understanding of folic acid and the impact that this knowledge has on effective folic acid consumption. Ultimately, the findings are hoped to contribute to improving effective folic acid intake rates through these investigations.

## Methods

**Ethics statement:** Obtaining informed consent was exempted by the Institutional Review Board of Seoul National University (No. 2109/001-014) because there was no sensitive information and the survey was anonymously treated.

### Study design

This cross-sectional survey study sought to examine the intake of folic acid and knowledge about folic acid among pregnant women in China and explore the influence of folic acid knowledge on effective folic acid intake.

### Participants

This study included women who were at least 12 weeks pregnant and were patients at Yantai Yuhuangding Hospital in Shandong, China. The participants were selected through convenience sampling. The study was based on previous research, which found that 42% of participants had taken folic acid prior to pregnancy [23]. An odds ratio (OR) of 2.64 was established, with a confidence level of 0.05 and a test power of 0.8, using G*power 3.1. The minimum sample size was calculated to be 134, but we anticipated a dropout rate of 20%, so the final sample size was set at 161. After data collection, it was found that a total of 154 women had completed the survey. However, after excluding 10 participants who did not complete the survey faithfully and four who had not taken folic acid, the total number of participants used in the survey analysis was 140.

### Instruments

After preparing the questionnaire and consent form in Korean, the researchers enlisted the help of a specialized agency for translation and reverse translation. This resulted in the final version of the tool, which was written in Chinese. The validity of this final questionnaire was subsequently assessed by two nursing educators who were proficient in both Chinese and Korean. Furthermore, a preliminary survey was carried out with three pregnant women living in China and no difficulties in understanding or completing the survey were found. Thus, items were deemed satisfactory for the main survey.

#### Effective folic acid intake

Effective folic acid intake was assessed based on the following criteria: (a) whether the daily intake of folic acid was 400 µg (yes/no), (b) whether folic acid was consumed starting from 1 month prior to pregnancy (yes/no), (c) whether folic acid was consumed during the first 3 months of pregnancy (yes/no), and (d) whether folic acid was consumed at least 24 days in a month (yes/no). A total score of 4 points indicated effective intake, while a score ranging from 0 to 3 points was considered indicative of ineffective intake.

#### Folic acid knowledge

We utilized nine items from the CDC’s 10-item questionnaire related to folic acid knowledge (https://www.cdc.gov/ncbddd/folicacid/quiz.html), after making necessary revisions. Each item was answered with either a “yes” or “no,” with correct responses earning 1 point and incorrect responses earning 0 points. The total score, which could range from 0 to 9, served as an indicator of the respondent’s knowledge of folic acid; a higher score signified greater knowledge. The Cronbach’s α for this knowledge tool is .65.

#### Folic acid intake

A questionnaire was developed based on Bai et al.’s study [24]. It comprised 18 items, which included questions about whether the respondent had taken folic acid, the first and last instances of folic acid intake, the total daily amount of folic acid consumed, and the specific content of the folic acid taken.

#### General characteristics

The general characteristics of the participants were collected using eight items: age, ethnicity, city residency, education level, spouse’s education level, occupation, spouse’s occupation, and monthly family income. The obstetric characteristics of the participants were as follows: the date of their last menstrual period, history of miscarriage, history of abortion, history of infertility, and whether the pregnancy was planned.

### Data collection

Data were collected from November 2021 to May 2022. A research assistant, who had previously worked as a nurse in China and had undergone training with the researchers, was responsible for data collection at Yantai Yuhungding Hospital in Shandong, China. Participants were recruited by scanning a QR code on a flyer posted in the hospital, which led them to the study’s information sheet. If a participant agreed to take part in the study, she proceeded to complete the online questionnaire. The information sheet outlined the study’s content, purpose, and data anonymity, and it also stated that participants could withdraw at any point during the study if they chose not to continue. If a participant clicked “do not agree,” the survey automatically ended. Completing the online questionnaire took approximately 10 minutes. As a token of appreciation, each participant received a mobile coffee coupon worth 3 US dollars.

### Data analysis

Data were analyzed as follows using IBM SPSS for Windows vers. 24.0 (IBM Corp., Armonk, NY, USA). The general and obstetric characteristics of the pregnant women were analyzed using frequency, percentages, mean, and standard deviation. Differences in effective folic acid intake according to folic acid knowledge were analyzed using the t-test and the chi-square test. Finally, logistic regression analysis was conducted to identify factors influencing folic acid intake.

## Results

### Characteristics of participants

The mean age of the 140 participants was 31.56±3.88 years (range, 21–41 years). In terms of education, 67.9% of the participants held university degrees, and 89.3% were employed. Among the participants’ spouses, 67.1% held university degrees, and 97.9% were employed.

Sixty percent of the participants were experiencing their first pregnancy. A smaller portion (8.6%) had previously experienced a miscarriage. Additionally, 30.7% had undergone an abortion. Notably, 82.9% of all participants had planned their pregnancies (Table 1).

### Status of effective folic acid intake

**Figure 1.**
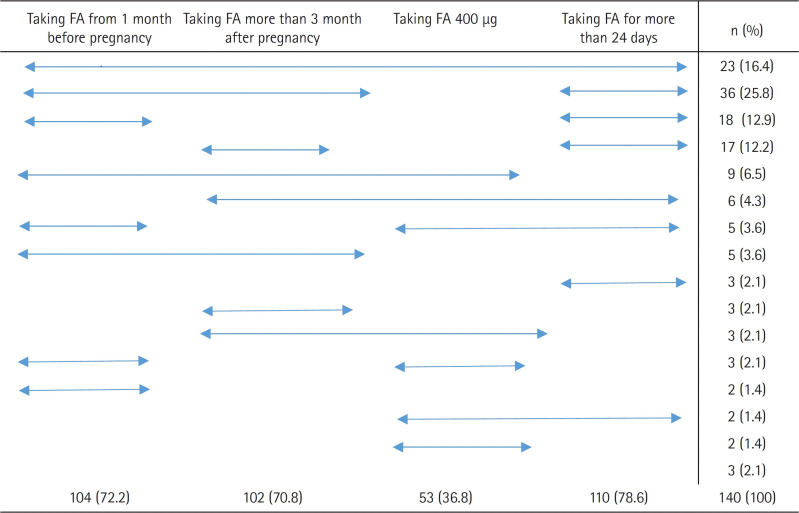
Folic acid (FA) intake status (N=140). Multiple responses were possible for the study participants regarding the status of FA intake, and three of the respondents did not comply with the conditions for effective folic acid intake even though they consumed FA.

Of the total participants, 16.4% (23 individuals) met all four criteria for effective folic acid intake, while the remaining 83.6% (117 individuals) did not. When we analyzed the intake outcomes based on these four criteria, we found that (1) 72.2% (104 individuals) began taking folic acid prior to pregnancy, (2) 70.8% (102 individuals) continued taking folic acid up to 3 months post-pregnancy, (3) only 36.8% (53 individuals) consumed the recommended daily dose of 400 µg of folic acid, and (4) 78.6% (110 individuals) took the supplement for at least 24 days in a month, which equates to 80% of the month. As these figures indicate, the criterion with the lowest adherence was the correct daily dosage of folic acid (Figure 1).

Among the participants who did not meet the criteria for effective folic acid intake, 25.8% (36 individuals) consumed folic acid from 1 month prior to pregnancy until 3 months post-pregnancy, for at least 24 days each month. This group represented the majority. They were followed by 12.9% (18 individuals) who consumed folic acid from before pregnancy for at least 24 days each month, 12.2% (17 individuals) who consumed folic acid until 3 months post-pregnancy for at least 24 days each month, and 6.5% (nine individuals) who consumed 400 µg of folic acid daily from before pregnancy until 3 months post-pregnancy. Among the participants with ineffective intake who underwent surgery, 2.1% (three individuals) did not meet any of the four criteria for effective folic acid intake.

### Differences in folic acid knowledge according to effective folic acid intake

The mean score for participants’ knowledge of folic acid was relatively high, at 5.61±2.18 out of a possible 9 points. The statement that received the highest rate of correct responses was “the easiest way to get the right amount of folic acid every day is to take 400 µg of synthetic folic acid,” with 79.3% (111 individuals) of participants responding “yes.” Conversely, the statement with the lowest rate of correct responses was “folic acid is a B vitamin,” with only 50.7% (71 individuals) of participants responding “yes.”

Upon analyzing the variance in folic acid knowledge based on effective folic acid intake, it was found that the group with effective folic acid intake scored higher in folic acid knowledge compared to the group with ineffective folic acid intake (t=4.10, *p*<.001). Among the items related to folic acid knowledge, participants who were aware that “women of childbearing age should consume 400 µg of folic acid every day” (χ^2^=10.95, *p*<.001), understood “ways to be sure that you are getting enough folic acid every day” (χ^2^=10.71, *p*<.001), knew “what are spina bifida and anencephaly” (χ^2^=3.98, *p*<.005), and recognized that “the easiest way to get the right amount of folic acid every day is to take 400 µg of synthetic folic acid” (χ^2^=4.49, *p*<.005), were more likely to exhibit effective folic acid intake (Table 2).

### Influencing factors on effective folic acid intake

To identify the factors that influence the effective intake of folic acid among pregnant women, we conducted a binomial logistic regression analysis. This analysis used significant variables derived from a cross-tabulation analysis, which examined potential differences based on general and obstetrical characteristics. However, our analysis revealed that these characteristics did not significantly impact the effective intake of folic acid among pregnant women (Table 1). We found that a higher score in folic acid knowledge (OR, 1.74; 95% confidence interval [CI], 1.29–2.35) was associated with effective folic acid intake. Notably, participants who understood that “women of childbearing age should consume 400 µg of folic acid daily” (OR, 14.77; 95% CI, 1.93–113.35) and knew “ways to be sure that you are getting enough folic acid every day” (OR, 5.74; 95% CI, 1.84–17.90) were more likely to effectively consume folic acid. However, when we adjusted the ORs using the characteristics found to be significant in effective folic acid intake, we did not identify any significant determinants (Table 3).

## Discussion

This study is the first, to our knowledge, to examine the status of effective folic acid intake and investigate its relationship with folic acid knowledge, in line with the four criteria set by the CDC’s folic acid intake guidelines. Our findings indicate that the rate of effective folic acid intake, based on these four guidelines, was 16.4%. This is significantly higher than the 4.82% rate of effective folic acid intake among pregnant women in China, as reported in a 2011 study. In that study, women consumed 400 µg of folic acid at least 5 days a week, starting 1 month before pregnancy and continuing until 2 months after conception [24]. This increase may be attributed to an actual rise in folic acid intake among pregnant women in China, a result of a policy that provided folic acid free of charge to boost intake rates [25]. However, the rate of effective intake remains relatively low.

This study can be compared to others that only adopted some of the criteria for effective folic acid intake. In 2017, the rate of effective folic acid intake, defined as consumption from 3 months before pregnancy until 3 months after pregnancy, was examined in Tianjin City. The rates of folic acid intake were found to be 14.4%, respectively [26]. In 2014, the rate of effective folic acid intake, defined as consumption from 3 months before pregnancy for at least 24 days a month, was analyzed in Shanxi Province. The rate of folic acid intake was found to be 14% [6], a figure similar to the intake rate in this study. All participants had taken folic acid at some point, but only a small proportion continued to take it from before conception through pregnancy. This finding is consistent with a study conducted overseas [27] and suggests that the participants’ lack of knowledge about the precise period for folic acid intake contributes to the low rate of effective folic acid intake.

In this study, 72.2% of participants reported taking folic acid for at least 1 month prior to pregnancy. This is significantly higher than the 24.7% rate reported in Cawley et al.’s study [13] involving pregnant women. To determine if there is a correlation between a participant’s level of education and the rate of folic acid intake, further studies are needed. This aligns with a previous study suggesting that a participant’s education level influences the rate of folic acid intake before pregnancy [26]. In this study, it was observed that 70.8% of participants continued taking folic acid up to 3 months post-pregnancy. This rate is comparable to the 66.1% reported in a study conducted with pregnant women in China [16]. This suggests that the “Health Guidelines for Preconception and during Pregnancy,” a public initiative launched in China in 2011 [28], has successfully raised awareness about the importance of folic acid intake among women. Consequently, most women now understand the need to take folic acid during pregnancy. Approximately 36.8% of the women in this study reported taking a daily dose of 400 µg of folic acid, a rate similar to the 47.2% reported in a study conducted with women in Italy [15]. This suggests that over half of the participants are unaware of the recommended daily dose of folic acid. According to a study by Maraschini et al. [15], only 8.0% of all participants received education on the correct dosage of folic acid prior to pregnancy, and 13.7% received such education during pregnancy. This underscores the fact that the importance of the correct dosage is not sufficiently emphasized in education about folic acid intake. Among the participants in this study, 78.6% reported taking folic acid for at least 24 days each month. This rate is similar to the 81.6% reported in a previous study conducted in China [23]. This suggests that the majority of women consistently take folic acid daily once they start.

In summary, while pregnant women in China were cognizant of the need to take folic acid up to 3 months post-pregnancy, and understood the frequency of its intake, they did not meet the recommended daily intake of 400 µg, starting at least 1 month prior to pregnancy. For effective folic acid consumption, individuals must strive to meet all these criteria in their daily routines. To boost the prevalence of effective folic acid consumption, several countries, including the United States, have instituted and enforced folic acid fortification policies. These policies promote the consumption of grain products fortified with folic acid. However, according to a meta-analysis by Toivonen et al. [29], there is no discernible difference in the incidence of neural tube defects between countries that have implemented folic acid fortification policies and those that have not. While county-level policies are necessary to increase the rate of effective folic acid intake, it appears that individual awareness of the importance of folic acid consumption, and the personal initiative to include it in one’s diet, are even more critical.

Turning to knowledge, the mean score for folic acid knowledge among the pregnant women participating in the study was 5.61 out of a possible 9 points. Two items had a correct response rate of 70% or higher, three items had a rate of 60% or higher, and four items had a rate of 50% or higher. This indicates that the overall understanding of folic acid is relatively high among the participants. However, further education is necessary on the items that scored lower to ensure accurate comprehension. These items included: “folic acid is a B vitamin,” “ways to be sure that you are getting enough folic acid every day,” “what are spina bifida and anencephaly,” and “a woman should be taking folic acid if she is planning a pregnancy, is capable of becoming pregnancy, even if she is not planning a pregnancy, or thinks she might become pregnant sometime in the future.” Additionally, the statement “women of childbearing age should consume 400 µg of folic acid daily” did not receive a high rate of correct responses. Given that this item also had the lowest adherence rate in the folic acid intake survey conducted as part of this study, there is a clear need for increased awareness and emphasis on this point.

The binomial logistic analysis revealed that participants with a more comprehensive understanding of folic acid were more likely to exhibit effective folic acid intake. This finding aligns with a survey conducted among pregnant women and those who had recently given birth in China. In this survey, women who understood the importance of folic acid during pregnancy and its role in preventing neural tube defects showed better adherence to folic acid intake [30]. Consequently, it is crucial to enhance folic acid knowledge to increase the rate of effective folic acid intake. The role of nurses, who are often the primary source of this knowledge, is also of paramount importance. Research indicates that the rate of folic acid intake among pregnant women and women of childbearing age increases when clinical staff provide brief, 30 to 60-second explanations about folic acid [31]. Therefore, nurses, who are tasked with educating these women, should emphasize the correct method of folic acid intake and instruct on how to effectively incorporate it into their routine.

In this study, factors such as the participant’s monthly income, education level, employment status, multiparity, miscarriage history, and whether the pregnancy was planned did not significantly influence effective folic acid intake. This finding contrasts with the study by Kim et al. [12] in Korea, which found that higher income, higher education level, and employment were associated with increased folic acid intake. It also differs from the study by Nilsen et al. [11] in Italy, which reported that primiparous women had a higher rate of effective folic acid intake. Specifically, our study found that whether a pregnancy was planned did not impact effective folic acid intake. This is in contrast to findings from Ireland, where women with planned pregnancies had a higher rate of folic acid intake [13]. The discrepancy may be due to the small sample size of our study and the fact that the majority of respondents reported having planned pregnancies, leading to oversampling. Furthermore, our study applied strict criteria for measuring effective intake, as opposed to the simple confirmation of intake used in previous studies. This could also account for the observed differences. In conclusion, the significant correlation between demographic and obstetrical characteristics and folic acid intake reported in previous studies was not observed in our study. This may be due to our smaller sample size, uneven distribution of demographic and obstetrical characteristics, and different method of measuring effective folic acid intake. Therefore, the relationship between demographic and obstetrical characteristics, including planned pregnancies, and effective folic acid intake should be re-evaluated in future studies with larger sample sizes.

The limitations of this study include the absence of a nationwide sample because it focused only on one specific area in China. In addition, it considered all four criteria of effective intake, making it difficult to compare the study directly with existing studies.

Nonetheless, this study provides meaningful results because it analyzed all four criteria of effective folic acid intake, pinpointing the specific reasons for unsuccessful implementation. Moreover, the study illustrates that to enhance the rate of effective folic acid intake, women of childbearing age need to cultivate an interest in folic acid consumption, a preventive strategy against neural tube defects. It underscores the necessity for comprehensive education on the precise dosage and duration of folic acid intake. We anticipate that this survey will offer participants the chance to proactively adopt effective folic acid intake practices in the future.

Based on the findings of this survey, future education on effective folic acid intake should take into account the following considerations. While most pregnant women are aware of the need to take folic acid, they may not be fully informed about the necessity of adhering to all four guidelines for effective intake, including its role in preventing neural tube defects. It is particularly important to emphasize that folic acid should be taken at least 1 month prior to pregnancy and that the recommended daily dosage for nonpregnant women is 400 µg.

There is an urgent need to educate women about folic acid, including its benefits and effective usage, for the betterment of women’s health. This education should be delivered in a clear and easily comprehensible manner. Future research should aim to validate the impact of this education on the understanding and effective consumption of folic acid. It should also reexamine the relationship between effective intake and demographic characteristics not previously investigated in this study. Furthermore, it appears necessary to monitor compliance with specific criteria for folic acid consumption through a mobile application.

## Figures and Tables

**Table 1.** Sociodemographic and obstetric characteristics and differences in effective folic acid intake according to general and obstetric characteristics (N=140)

Characteristics	Categories	Total (N=140), n (%)	Folic acid intake, n (%)		*χ^2^* (*p*)
			Effective^†^ (n=23)	Ineffective (n=117)	
*Sociodemographic characteristics*					
Age (year)	Mean±SD (range), 31.56±3.88 (24–41)				20.34 (.257)
	24–29	42 (30.0)	5 (21.7)	37 (31.6)	
	30–34	69 (49.3)	11 (47.9)	58 (49.6)	
	35–41	29 (20.7)	7 (30.4)	22 (18.8)	
Nationality	Han nationality	122 (87.1)	21 (91.3)	101 (86.3)	0.43 (.514)
	Minority nationality	18 (12.9)	2 (8.7)	16 (13.7)	
Location of residence	Town	128 (91.4)	21 (91.3)	107 (91.5)	0.00 (.981)
	Countryside	12 (8.6)	2 (8.7)	10 (8.5)	
Education	≤High school	45 (32.1)	6 (26.1)	39 (33.3)	0.46 (.496)
	≥College	95 (67.9)	17 (73.9)	78 (66.7)	
Husband’s education	≤High school	46 (32.9)	7 (30.4)	39 (33.3)	0.07 (.787)
	≥College	94 (67.1)	16 (69.6)	78 (66.7)	
Employment	Employed	125 (89.3)	21 (91.3)	104 (88.9)	0.12 (.732)
	Unemployed	15 (10.7)	2 (8.7)	13 (11.1)	
Husband’s employment	Employed	137 (97.9)	23 (100)	114 (97.4)	0.60 (.438)
	Unemployed	3 (2.1)	0 (0)	3 (2.6)	
Monthly income (Chinese yuan^‡^)	≤10,000	76 (54.3)	15 (65.2)	61 (52.1)	1.33 (.250)
	>10,000	64 (45.7)	8 (34.8)	56 (47.9)	
*Obstetric characteristics*					
Parity	Primiparous	84 (60.0)	17 (73.9)	67 (57.3)	2.22 (.136)
	Multiparous	56 (40.0)	6 (26.1)	50 (42.7)	
History of miscarriage	Yes	12 (8.6)	3 (13.0)	9 (7.7)	2.45 (.294)
	No	128 (91.4)	20 (87.0)	108 (92.3)	
History of abortion	Yes	43 (30.7)	15 (65.2)	82 (70.1)	0.21 (.644)
	No	97 (69.3)	8 (34.8)	35 (29.9)	
History of infertility	Yes	10 (7.1)	3 (13.0)	7 (6.0)	1.45 (.229)
	No	130 (92.9)	20 (87.0)	110 (94.0)	
Planned pregnancy	Yes	116 (82.9)	22 (95.7)	94 (80.3)	3.17 (.075)
	No	24 (17.1)	1 (4.3)	23 (19.7)	

† Effective folic acid intake is 400 μg of folic acid taken more than 24 days every month from 1 month preconception until 3 months after pregnancy.

‡ 10,000 Chinese yuan is roughly 1,380 US dollars.

**Table 2.** Differences in effective folic acid intake according to correct folic acid knowledge (N=140)

Question	Total (N=140), n (%)	Folic acid intake, n (%)		χ^2^ or t (*p*)
		Effective ^†^ (n=23)	Ineffective (n=117)	
1. Folic acid is a B vitamin	71 (50.7)	15 (65.2)	56 (47.9)	2.32 (.128)
2. Folic acid reduces the risk for spina bifida and anencephaly	100 (71.4)	20 (87.0)	80 (68.4)	3.25 (.071)
3. Women of childbearing age should consume 400 µg of folic acid every day	92 (65.7)	22 (95.7)	70 (59.8)	10.95 (<.001)
4. Ways to be sure that you are getting enough folic acid every day	72 (51.4)	19 (82.6)	53 (45.3)	10.71 (<.001)
5. What are spina bifida and anencephaly	77 (55.0)	17 (73.9)	60 (51.3)	3.98 (.046)
6. In order to help prevent neural tube defects, folic acid must be taken before and during the first few months of pregnancy	93 (66.4)	17 (73.9)	76 (65.0)	0.69 (.406)
7. While it’s important to eat a healthy diet, the easiest way to get the right amount of folic acid every day is to take 400 µg of synthetic folic acid	111 (79.3)	22 (95.7)	89 (76.1)	4.49 (.034)
8. A woman should be talking folic acid if she is planning a pregnancy, is capable of becoming pregnant, even if she is not planning a pregnancy, or thinks she might become pregnant sometime in the future	72 (51.4)	16 (69.6)	56 (47.9)	3.62 (.057)
9. Now that you are an expert on folic acid, you should make sure to take a multivitamin containing folic acid every day, eat foods rich in folate, and foods fortified with synthetic folic acid, tell a friend about the importance of folic acid	97 (69.3)	18 (78.2)	79 (67.5)	1.04 (.307)
Total score (possible range, 0–9), mean±SD	5.61±2.18	7.22±1.65	5.29±2.13	4.10 (<.001)

† Effective folic acid intake is 400 µg of folic acid taken more than 24 days every month from 1 month preconception until 3 months after pregnancy.

**Table 3.** Effect of folic acid knowledge on effective folic acid intake (N=140)

Variable	Categories (%)	Effective folic acid intake^†^	
		Unadjusted OR (95% CI)	Adjusted OR (95% CI)
Monthly income (Chinese yuan^‡^)	≤10,000 (53.5)	0.58 (0.23–1.48)	-
	>10,000 (46.5)	Reference	-
Education	College (68.1)	1.42 (0.58–3.88)	-
	High school (31.9)	Reference	-
Employment	Employed (88.9)	1.31 (0.28–6.25)	-
	Unemployed (11.1)	Reference	-
Parity	Multiparous (40.3)	0.47 (0.17–1.29)	-
	Primiparous (59.7)	Reference	-
Abortion	Yes (29.9)	1.25 (0.49–3.22)	-
	No (70.1)	Reference	-
Planned pregnancy	Yes (81.9)	0.19 (0.02–1.45)	-
	No (18.1)	Reference	-
Knowledge score (0–9)		1.74^§^ (1.29–2.35)	1.74 (0.51–1.88)
3. Women of childbearing age should consume 400 µg of folic acid every day	Yes (65.7)	14.77^§^ (1.93–113.35)	1.76 (0.79–1.27)
	No (34.3)	Reference	-
4. Ways to be sure that you are getting enough folic acid every day	Yes (51.4)	5.74^§^ (1.84–17.90)	1.04 (0.60–1.66)
	No (48.6)	Reference	-
5. What are spina bifida and anencephaly	Yes (55.0)	2.69 (0.99–7.31)	-
	No (45.0)	Reference	-
7. While it’s important to eat healthy diet, the easiest way to get the right amount of folic acid every day is to take 400 µg of synthetic folic acid	Yes (79.3)	6.92 (0.89–53.69)	-
	No (20.7)	Reference	-

CI, confidence interval; OR, odds ratio.

† Effective folic acid intake is 400 µg of folic acid taken more than 24 days every month from 1 month preconception until 3 months after pregnancy.

‡ 10,000 Chinese yuan is roughly 1,380 US dollars.

§ *p*<.05.

## References

[1] Centers for Disease Control and Prevention Folic acid recommendations [Internet]. https://www.cdc.gov/ncbddd/folicacid/recommendations.html.

[2] Zaganjor I, Sekkarie A, Tsang BL, Williams J, Razzaghi H, Mulinare J (2016). Describing the prevalence of neural tube defects worldwide: a systematic literature review. PLoS One.

[3] Kong YM, Xiang K, Luo Y, Zhu BS (2015). Regional difference in the incidence of the morbidity of neural tube defects in China and intervention strategies. Chin J Pract Gynecol Obstet.

[4] Blount JP, George TM, Koueik J, Iskandar BJ (2019). Concepts in the neurosurgical care of patients with spinal neural tube defects: an embryologic approach. Birth Defects Res.

[5] Chitayat D, Matsui D, Amitai Y, Kennedy D, Vohra S, Rieder M (2016). Folic acid supplementation for pregnant women and those planning pregnancy: 2015 update. J Clin Pharmacol.

[6] Yang S, Liu Y, Dang W, Zhang X (2018). Statue of folic acid intake of the urban pregnant women of Shanxi province and its influence factors. Chin J Fam Plann.

[7] Ren XF, Zhang L (2017). [Analysis of 1635 cases of women taking fo­lic acid in early pregnancy in areas with high birth defects in Shaanxi Province and its influencing factors]. Chin J Reprod Heatlth.

[8] Yang S, Jin YS, Zhang XJ (2018). [Current status of folic acid knowledge, beliefs, and practices among rural pregnant women in Shanxi Province and its influencing factors]. Chin J Reprod Health.

[9] Li F, Lin Q, Hu XY, Li MZ, Qin H, Yang LN (2014). The current situation of folic acid supplementation and its influencing factors among rural women of child-bearing age in Changsha County. Pract Prev Med.

[10] Yamamoto S, Wada Y (2018). Awareness, use and information sources of folic acid supplementation to prevent neural tube defects in pregnant Japanese women. Public Health Nutr.

[11] Nilsen RM, Leoncini E, Gastaldi P, Allegri V, Agostino R, Faravelli F (2016). Prevalence and determinants of preconception folic acid use: an Italian multicenter survey. Ital J Pediatr.

[12] Kim J, Yon M, Kim CI, Lee Y, Moon GI, Hong J (2017). Preconceptional use of folic acid and knowledge about folic acid among low-income pregnant women in Korea. Nutr Res Pract.

[13] Cawley S, Mullaney L, McKeating A, Farren M, McCartney D, Turner MJ (2016). An analysis of folic acid supplementation in women presenting for antenatal care. J Public Health (Oxf).

[14] Bixenstine PJ, Cheng TL, Cheng D, Connor KA, Mistry KB (2015). Association between preconception counseling and folic acid supplementation before pregnancy and reasons for non-use. Matern Child Health J.

[15] Maraschini A, D'Aloja P, Lega I, Buoncristiano M, Kirchmayer U, Ventura M (2017). Do Italian pregnant women use periconceptional folate supplementation?. Ann Ist Super Sanita.

[16] Guo HJ, Wang XG, Guo Y (2014). [Analysis on knowledge and be­havior of folic acid supplementation among 431 pregnant women]. Matern Child Health Care China.

[17] Cawley S, Mullaney L, McKeating A, Farren M, McCartney D, Turner MJ (2016). Knowledge about folic acid supplementation in women presenting for antenatal care. Eur J Clin Nutr.

[18] Poels M, van Stel HF, Franx A, Koster MP (2017). Actively preparing for pregnancy is associated with healthier lifestyle of women during the preconception period. Midwifery.

[19] Hao N, Xia W, Tang Y, Wu M, Jiang H, Lin X (2015). Periconceptional folic acid supplementation among pregnant women with epilepsy in a developing country: a retroprospective survey in China. Epilepsy Behav.

[20] Kim MJ, Kim J, Hwang EJ, Song Y, Kim H, Hyun T (2018). Awareness, knowledge, and use of folic acid among non-pregnant Korean women of childbearing age. Nutr Res Pract.

[21] Obara T, Nishigori H, Nishigori T, Metoki H, Ishikuro M, Tatsuta N (2017). Prevalence and determinants of inadequate use of folic acid supplementation in Japanese pregnant women: the Japan Environment and Children's Study (JECS). J Matern Fetal Neonatal Med.

[22] Goshu YA, Liyeh TM, Ayele AS, Zeleke LB, Kassie YT (2018). Women’s awareness and associated factors on preconception folic acid supplementation in Adet, Northwestern Ethiopia, 2016: implication of reproductive health. J Nutr Metab.

[23] Li D, Huang L, Yang W, Qi C, Shang L, Xin J (2019). Knowledge, attitude and practice level of women at the periconceptional period: a cross-sectional study in Shaanxi China. BMC Pregnancy Childbirth.

[24] Bai YN, Zhu J, Wang MZ, Cheng N, Hu XB, Du WQ (2011). [Current status of folic acid compliance behavior among pregnant women in Gansu province and a study on the definition of folic acid compliance]. J Hyg Res.

[25] Ji GP (2017). [Effect evaluation of folic acid supplementation program for prevention of neural tube defects in Anhui Province]. Matern Child Health Care China.

[26] Yan J, Zheng YZ, Cao LJ, Liu YY, Li W, Huang GW (2017). Periconceptional folic acid supplementation in Chinese women: a cross-sectional study. Biomed Environ Sci.

[27] McNulty B, Pentieva K, Marshall B, Ward M, Molloy AM, Scott JM (2011). Women's compliance with current folic acid recommendations and achievement of optimal vitamin status for preventing neural tube defects. Hum Reprod.

[28] Chinese Society of Obstetrics and Gynecology, Chinese Medical Association (2011). Guideline for prenatal care and antenatal care (edition 1). Chin J Obstet Gynecol.

[29] Toivonen KI, Lacroix E, Flynn M, Ronksley PE, Oinonen KA, Metcalfe A (2018). Folic acid supplementation during the preconception period: a systematic review and meta-analysis. Prev Med.

[30] Wu T, Dang S (2019). Effect of folic acid supplementation-related cognition on folic acid intake among childbearing women in Shaanxi province: a log-binomial regression model analysis. Chin J Public Health.

[31] Robbins JM, Cleves MA, Collins HB, Andrews N, Smith LN, Hobbs CA (2005). Randomized trial of a physician-based intervention to increase the use of folic acid supplements among women. Am J Obstet Gynecol.

